# Adalimumab induced exacerbation of psoriasis in patients with combined pemphigus: A case report

**DOI:** 10.1097/MD.0000000000036988

**Published:** 2024-01-26

**Authors:** Limin Yao, Xiaoli Cai, Xiaoqing Du, Yuxin Ma

**Affiliations:** a Department of Dermatology, Bethune International Peace Hospital, Shijiazhuang, Hebei, China; b Department of Pathology, Bethune International Peace Hospital, Shijiazhuang, Hebei, China.

**Keywords:** adalimumab, case report, pemphigus, psoriasis

## Abstract

**Rationale:**

Psoriasis is an immune-related disease caused by genetic factors, abnormalities in the immune system and environmental factors, while pemphigus is an autoimmune disease caused by the autoimmune system attacking the skin and mucosal tissues. Herein, we aimed to report a rare case of adalimumab induced exacerbation of psoriasis patients with pemphigus. The rare disease causes considerable challenges for clinical diagnosis and treatment.

**Patient concerns:**

The patient was a 43-year-old man with intermittent erythema and scaling all over the body for more than 20 years, and blisters and vesicles on the trunk and limbs for 1 month. Half a year ago, the patient had blisters on the limbs, and was diagnosed with deciduous pemphigus in a hospital, and the blisters subsided after being given traditional Chinese medicine orally. Half a month ago, the erythema area was enlarged, and adalimumab 80 mg intramuscular injection was given for 1 time after consultation in the hospital. On the following day, the area of erythema and scales was suddenly enlarged obviously compared with the previous 1, and obvious blisters and vesicles appeared on the limbs, neck, and trunk, which were aggravated progressively and accompanied by obvious itching and pain.

**Diagnoses:**

The patient was diagnosed with psoriasis in patients with combined pemphigus.

**Intervention:**

After combined treatment with methylprednisolone and cyclosporine, the skin lesions have basically recovered.

**Outcomes:**

The skin lesions have basically healed. Follow up for 6 months without recurrence.

**Lessons:**

Methylprednisolone combined with cyclosporine may be an option in treating patients with psoriasis patients with pemphigus.

## 1. Introduction

Psoriasis is a chronic skin disease characterized by the appearance of red, scaly plaques.^[1]^ The disease usually affects the skin, nails and joints and may cause itching and discomfort.^[2]^ Although the exact cause of psoriasis is unknown, it is thought to be linked to genetics, immune system abnormalities, and environmental factors.^[3]^ Pemphigus, on the other hand, is a group of autoimmune disorders that includes several types, such as pemphigus-like epidermolysis bullosa.^[4]^ The common feature of these diseases is the development of blisters, herpes, and ulcers on the skin and mucous membranes as a result of the immune system attacking normal intercellular adhesion proteins or adhesion structures. In this article, we report a rare case of adalimumab induced exacerbation of psoriasis patients with pemphigus.

## 2. Case presentation

Patient is a 43-year-old male presenting with intermittent erythematous plaques and scales throughout his body for over 20 years. He developed bullae and erosions on his trunk and extremities 1 month ago. About 20 years ago, he had no apparent trigger, but gradually developed erythematous plaques and scales on his extremities and trunk intermittently without clear seasonal variations. He self-treated with topical medications (specifics unknown), and the condition remained stable. Six months ago, he developed bullae on his extremities, diagnosed with pemphigus foliaceus in another hospital, and treated with traditional oral Chinese medicine. The bullae resolved after treatment. Two weeks ago, the erythematous plaques expanded, and the patient presented to our clinic where he was diagnosed with psoriasis. Adalimumab 80 mg was administered once by intramuscular injection, and the next day the area of erythematous plaques and scales increased markedly compared to before. The patient developed significant pruritus and pain along with obvious bullae and erosions on his extremities, neck, and trunk, which gradually worsened. The diagnosis was unclear between psoriasis and pemphigus foliaceus, and the patient was admitted to the hospital.

**Figure 1.**
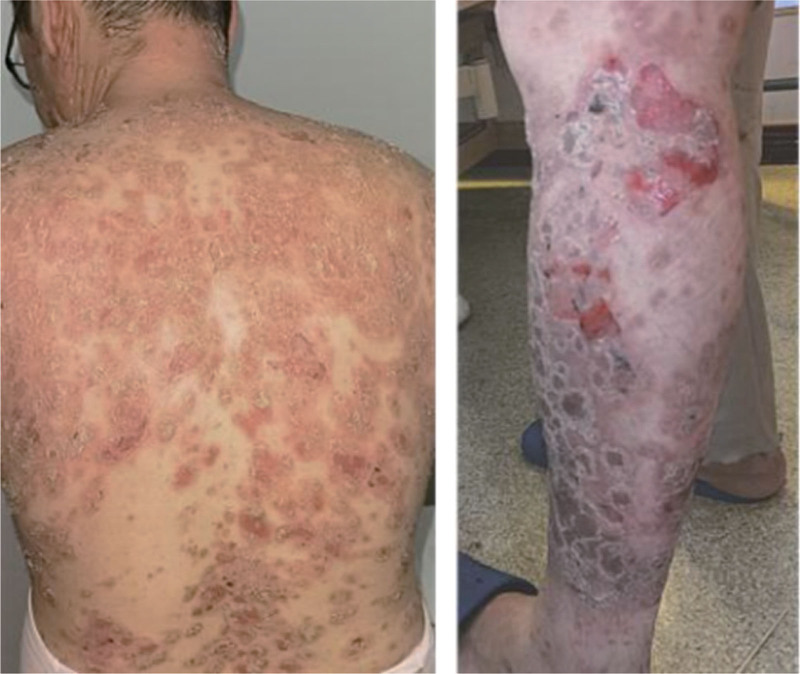
Patients before treatment (back and lower limbs).

**Figure 2.**
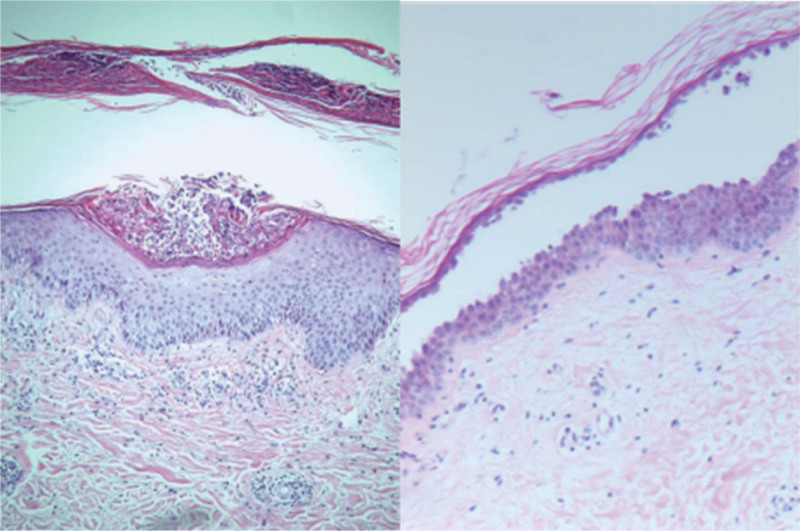
Hematoxylin and eosin, original magnification 10×.

The patient has a past medical history, personal history, and family history with no particular issues. There were no abnormalities found during physical examination. Dermatological findings show large erythematous plaques on the head, face, trunk, and extremities covered with silver-white or yellow-white scales. In some areas of edematous erythema on the trunk and extremities, there are many bullae and erosions with exudate. The bullae have a loose wall and clear fluid, with positive Nikolsky sign. Some bullae have dried, leaving thick honey-colored crusts (Fig. 1). The bacterial analysis of secretion material smear showed *Staphylococcus aureus*. The autoimmune series for pemphigus panel was as follows: desmoglein-1 (Dsg1) antibody: 139.6 U/mL (normal range: 0–20 U/mL), desmoglein-3 (Dsg3) antibody: 2 U/mL (normal range: 0–20 U/mL), and bullous pemphigoid antigen 180 (BP180): 0.5 U/mL (normal range: 0–9 U/mL). The pathological examination on the red plaque on the back showed hyperkeratosis and incomplete keratinization of the epidermis, with microabscesses in the stratum corneum and psoriasis-like epidermal hyperplasia. The pathological examination on the bullae lesion revealed partial loss of the epidermis, focal acantholysis, and bullae in the superficial layer of the epidermis, and perivascular lymphocyte infiltration in the superficial layer of the dermis (Fig. 2). The diagnosis is psoriasis with pemphigus foliaceus.

**Figure 3.**
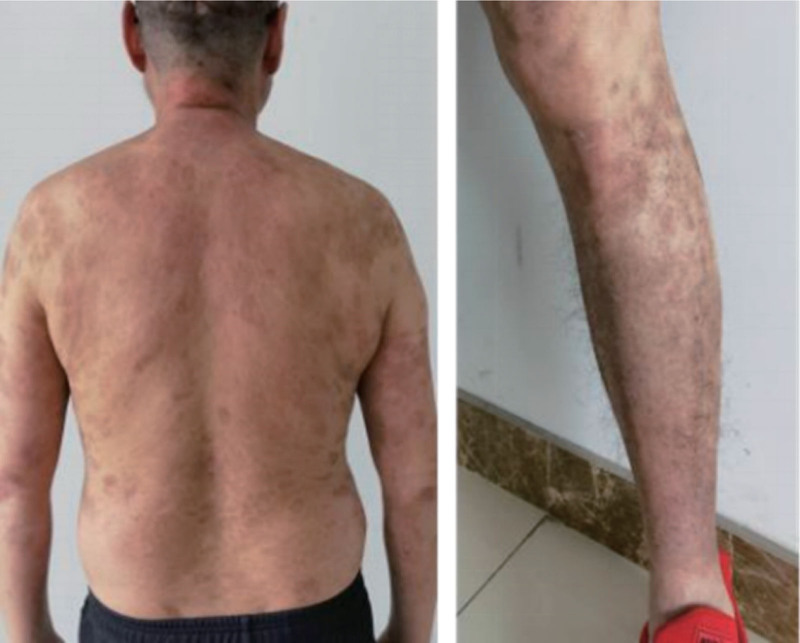
After therapy with methylprednisolone and cyclosporine (back and lower limbs).

Based on the clinical presentation, serological, and pathological examination, the patient was diagnosed with psoriasis with pemphigus foliaceus. Upon admission, the patient was treated with methylprednisolone sodium succinate 40 mg qd and avise gelatin capsule 20 mg qd. However, on the 4th day, erythema continued to expand, and new bullae and erosions appeared daily, accompanied by an increased white blood cell count. The treatment plan was adjusted to methylprednisolone sodium succinate 60 mg qd, methotrexate injection 5 mg qw, and ceftriaxone sodium 2 g bid. After 5 days, the patient’s liver function became abnormal, so methotrexate and avise were discontinued, and cyclosporine 100 mg bid was added. The patient’s condition was well-controlled, the area of erythema did not increase, there were no new bullae, and the edematous erythema on the extremities had subsided significantly. The bullae had dried and formed crusts. During hospitalization, the patient’s condition was well-controlled, and the original erythematous plaques and scales of psoriasis had significantly improved (Fig. 3). Two weeks after discharge, steroid dosage was reduced by 10%, but cyclosporine remained unchanged. Blood pressure, urine routine, liver and kidney function were monitored during medication use, and there were no significant abnormalities found. After 6 months of follow-up, the patient did not relapse.

## 3. Discussion and conclusion

Psoriasis is a chronic immune-related skin disease, and plaque psoriasis is the most common type with various comorbidities. In 1978, Koerber et al first reported some blistering diseases associated with psoriasis,^[5]^ with the most common being bullous pemphigoid, and there are fewer reports of pemphigus foliaceus associated with it.^[6]^ Studies have shown that the likelihood of psoriasis patients developing new-onset pemphigus foliaceus is more than 3 times that of the control group. Additionally, among all published studies, the incidence of comorbid psoriasis in pemphigus foliaceus patients is 2.4%. The most common types of pemphigus foliaceus associated with psoriasis are pemphigus erythematosus, followed by pemphigus vulgaris, IgA pemphigus, and herpes-like pemphigus.^[7]^ Adalimumab is a fully humanized monoclonal antibody against tumor necrosis factor alpha, which can effectively inhibit tumor necrosis factor alpha-involved inflammation and can be used in the treatment of ankylosing spondylitis and psoriasis. In recent years, some antitumor necrosis factor biologics have been found to induce or exacerbate psoriasis when used to treat it.^[8,9]^ After adverse reactions occur, local or systemic medication can be used for treatment. This case report describes a case of adalimumab-induced psoriasis with pemphigus foliaceus exacerbation. The patient was treated with cyclosporine combined with glucocorticoids and gradually improved. Therefore, when using this class of drugs, close attention should be paid to the possible adverse reactions that may occur so that better handling measures can be taken.

However, this case report has limitations and is not universally applicable. The limited sample size and the need to further increase and expand the sample size. Further research on the efficacy of cyclosporine combined with glucocorticoids for adalimumab-induced psoriasis with pemphigus foliaceus exacerbation is still worth investigating in the future.

## Author contributions

**Conceptualization:** Limin Yao, Xiaoli Cai, Xiaoqing Du, Yuxin Ma.

**Data curation:** Limin Yao, Xiaoqing Du, Yuxin Ma.

**Formal analysis:** Xiaoli Cai, Xiaoqing Du.

**Funding acquisition:** Limin Yao, Xiaoqing Du.

**Investigation:** Xiaoli Cai, Yuxin Ma.

**Methodology:** Limin Yao, Xiaoqing Du.

**Project administration:** Xiaoli Cai.

**Resources:** Limin Yao, Yuxin Ma.

**Software:** Limin Yao.

**Supervision:** Xiaoli Cai.

**Validation:** Limin Yao, Xiaoqing Du.

**Visualization:** Limin Yao, Yuxin Ma.

**Writing – original draft:** Limin Yao.

**Writing – review & editing:** Limin Yao.
